# Strawberry Juice Powders: Effect of Spray-Drying Conditions on the Microencapsulation of Bioactive Components and Physicochemical Properties

**DOI:** 10.3390/molecules26185466

**Published:** 2021-09-08

**Authors:** César Leyva-Porras, María Zenaida Saavedra-Leos, Laura Araceli López-Martinez, Vicente Espinosa-Solis, Yolanda Terán-Figueroa, Alberto Toxqui-Terán, Isaac Compeán-Martínez

**Affiliations:** 1 Centro de Investigación de Materiales Avanzados (CIMAV), Complejo Industrial Chihuahua, Chihuahua 31136, Mexico; cesar.leyva@cimav.edu.mx; 2 Coordinación Académica Región Altiplano, Universidad Autónoma de San Luis Potosí, Matehuala, San Luis Potosí 78700, Mexico; zenaida.saavedra@uaslp.mx; 3 Coordinación Académica Región Altiplano Oeste, Universidad Autónoma de San Luis Potosí, Carretera Salinas-Santo Domingo 200, Salinas de Hidalgo, San Luis Potosí 78600, Mexico; araceli.lopez@uaslp.mx; 4 Coordinación Académica Región Huasteca Sur, Universidad Autónoma de San Luis Potosí, Carretera Tamazunchale-San Martin Km. 5. Tamazunchale, San Luis Potosí 79960, Mexico; vicente.espinosa@uaslp.mx; 5 Facultad de Enfermería y Nutrición, Universidad Autónoma de San Luis Potosí, Av. Dr. Manuel Nava 6, San Luis Potosí 78210, Mexico; yolandat@uaslp.mx; 6 Centro de Investigación de Materiales Avanzados (CIMAV), Parque de Investigación e Innovación Tecnológica, Apodaca, Nuevo León 66600, Mexico; alberto.toxqui@cimav.edu.mx

**Keywords:** carrying agent, maltodextrin, total phenolic content, thermal characterization

## Abstract

The drying of fruit juices has advantages such as easy handling of powders, reduction in volume, and preservation of the characteristics of the fruit. Thus, in this work, the effect of the spray drying conditions of strawberry juice (SJ) with maltodextrin (MX) as a carrying agent on the microencapsulation of bioactive compounds and physicochemical properties was studied. The content of phenolic compounds and antioxidant activity showed higher values at low concentrations of MX, while the effect of drying temperature was negligible. The thermal characterization showed that the low molecular weight sugars in the juice decreased the glass transition temperature (Tg). The morphological analysis by scanning electron microscopy (SEM) indicated that at low concentrations of MX, the particles agglomerated, while at intermediate and high concentrations, the particles were observed as well separated. Through microstructural analysis by X-ray diffraction (XRD), the presence of amorphous state was confirmed in all the samples, which is beneficial for preventing chemical and biochemical reactions, and promoting the conservation of the microencapsulated bioactive compounds.

## 1. Introduction

Strawberry (*Fragaria x ananassa* Duch.) is a fruit largely consumed as fresh or in prepared foods, with a characteristic aroma, red color, and sweet taste. In 2019, the world production of strawberries was 8.9 million tons, of which China contributed 35.9%, USA 11.2%, and Mexico 10.11% [1]. Strawberry is an appreciated fruit for its nutritional properties and claimed beneficial effects. These fruits are consumed in considerable quantities, either fresh or in prepared foods [2].

Strawberries are rich in nutritious compounds including sugars, organic acids, and vitamin C, as well as a wide range of phytochemicals, especially polyphenols, among which, phenolic acids, lignans and flavonoids containing anthocyanins, tannins, and flavonols, are the major phenolic compounds found in this fruit [3,4]. Some of the most representative and important flavonols in strawberry fruit are kaempferol and quercetin derivatives, while free ellagic acid and its derivatives are the representative ellagitannins. Phenolic compounds containing these flavonols and ellagitannins are well-known for their antioxidative activity potential, whereby they play an important role in plant protection against biotic and abiotic stress factors [3,4]. The color in strawberry fruit results from the content of this anthocyanins [5]. Strawberries have shown a remarkably high scavenging activity toward chemically generated radicals, thus making them effective in inhibiting oxidation of human low-density lipoproteins. Antioxidant activity of strawberries could contribute to the prevention of cancer, cardiovascular, and other chronic diseases [2].

Strawberry harvest is seasonal, while the fruit is perishable, extremely susceptible to mechanical damage, and easily spoiled. In this sense, different industrial methodologies have been employed to promote the conservation of the fruit. These technologies include the frozen of the fruit, the preparation of processed intermediate products, and drying processes. Once the fruit is dried, it can be used to produce several food products, such as food concentrates, desserts, fruit teas, conditioners, and dietary supplements [6]. The use of carrying agents in the spray drying is a fast and reliable methodology employed for removing the water content of fruit juices, increasing shelf life and stability of the dried product [7]. Through this methodology, bioactive molecules and temperature-sensitive ingredients are encapsulated within the carrier agent that acts as coating or protecting wall to isolate from the environmental conditions and oxidation [8]. However, the processing conditions such as concentration of the carrying agent, inlet temperature, and feed flow, may affect the final physicochemical properties of the dried product. For example, Saavedra-Leos et al. 2019 studied the spray drying conditions of blueberry juice [9]. They reported that a concentration of 25% of maltodextrin (MX) and processing temperature of 210 °C were the optimal conditions for obtaining the highest yield and content of 3-D-galactoside. Recently, Saavedra-Leos et al. 2021 also tested the drying conditions of broccoli juice on the conservation of antioxidants [10]. They found that the total phenolic content (TPC) and antioxidant activity (AA) were optimal at a concentration of 5% of MX and inlet temperature of 220 °C. In these works, maltodextrins have been employed as high molecular weight additives to increase the overall glass transition temperature of the product [11]. Although other carbohydrate polymers have been used as carrying agents in the drying of fruit juices such as inulin [12], whey protein isolate [13], and arabic gum [14], the performance of MX on the conservation of antioxidants has been outstanding [15].

Regarding strawberry, only few works have reported the spray drying of strawberry juice (SJ) and puree. For example, Sadowska et al. [6] reported the bioactive, physicochemical, and sensory properties, as well as the microstructure of strawberry juice powders obtained by different drying methods. Among the methods employed is spray drying (SD) with maltodextrin as drying aid at concentration of 60%. On the other hand, Gong et al. [13] evaluated the effect of mixing two carrier agents, whey protein isolate (WPI) and MX, with strawberry pure (S) by SD. They used 60% of S, 30–40% of MX, and 0–10% of WPI. They reported the physical and thermal stability of the powders obtained. Evidently, both works employed a high concentration of the carrying agent, which may affect the final quality of the dried product.

Therefore, it is necessary to test with low carrier agent contents and relatively lower drying temperatures. For this reason, the present work aims to establish the spray drying of strawberry juice (SJ) using low concentrations of MX (5, 7.5, and 10%) and low inlet temperatures (150, 185, and 220 °C). The microencapsulation of the bioactive compounds was tested on total phenolic content (TPC) and antioxidant activity (AA). Furthermore, the thermal, morphological, and microstructural properties of the powders were discussed. This work contributes to establish an alternative procedure for the spray drying of fruit juices, complex sugar-rich systems, and heat-sensitive compounds.

## 2. Results and Discussion

### 2.1. Total Phenolic Content and Antioxidant Activity

**Figure 1:**
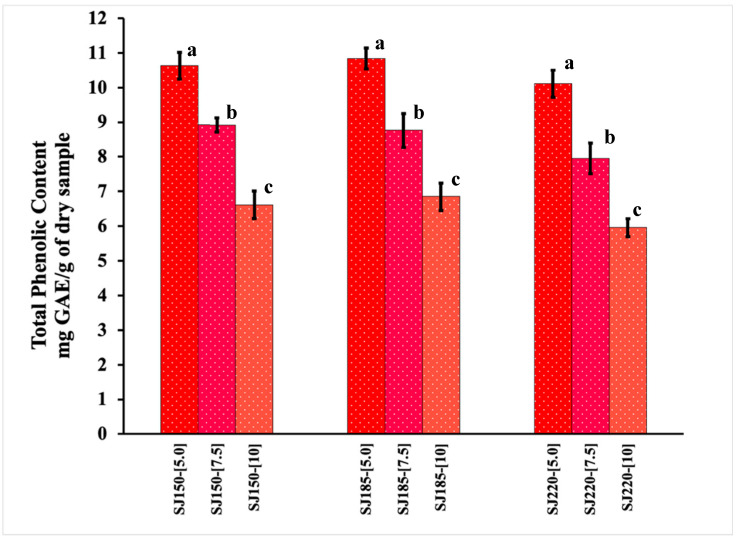
Total phenolic content of spray-dried strawberry juice-maltodextrin (SJ-MX) powders under different experimental conditions. Inlet Temperatures (150, 185 and 220 °C) and MX concentrations (5%, 7.5% and 10%). a, b and c are parameters of the Tukey’s multiple post hoc tests.

Figure 1 presents the TPC of the spray-dried strawberry juice-maltodextrin (SJ-MX) powders obtained at different inlet temperatures and MX concentrations. TPC values are expressed in terms of the mg of gallic acid equivalents (GAE) consumed per gram of dried SJ-MX. The results showed a statistically significant (*p* = 0.05) decrease of the TPC with the increment of MX concentration. The highest TPC values (10.8 ± 0.19 mg GAE/g dry sample) were recorded at the lowest MX concentration of 5%. While at the highest MX concentration (10%) presented, the lowest TPC values were observed (5.9 ± 0.33 mg GAE/g dry sample). On the other hand, the effect of the inlet temperature on the TPC was negligible; no statistically significant differences (*p* = 0.05) were found in SJ-MX powders at a same level of MX and different inlet temperature. For example, the TPC values at MX concentration of 5% were found in an interval of 10.1–10.8 mg GAE/g dry sample, at temperatures of 150–220 °C. A similar behavior was observed for the 7.5 and 10% MX concentration.

The TPC methodology is an indirect determination of the content of the phenolic compounds, which is based on the formation of a complex formed by phosphotungstates and molybdates, and the electron transfer observed as the blue coloration in the solution [16]. Nevertheless, the presence of non-phenolic compounds naturally present in juices and fruits, such as ascorbic acid, glucose, fructose, and sulfites, may influence the TPC [17]. Sadowska et al. [6] studied three methods for drying strawberry (*Fragaria x ananassa*) cultivar and determined the TPC by HPLC–UV/VIS. The TPC value for the fresh strawberry was 8.049 mg/g of the sample, while the value for the spray-dried sample was 1.29 mg/g of the sample. The drying conditions employed were a concentration of 60% of MX, and an inlet temperature of 160 °C. Evidently, the TPC values reported herein are relatively higher than those reported in literature for fresh and spray-dried strawberry, suggesting that the experimental conditions selected positively influenced the conservation of the phenolic compounds.

In addition to the phenolic compounds, strawberry contains different bioactive components that impart the characteristic red color of the fruit. More than 25 different anthocyanins have been reported in strawberry cultivars, the main three being: pelargonidin 3- O-glucoside, pelargonidin 3-O-rutinoside, and cyanidin 3-O-glucoside. Of these, pelargonidin 3-O-glucoside provides a bright red color, whereas cyanidin 3-O-glucoside gives a dark red color [5].

**Figure 2:**
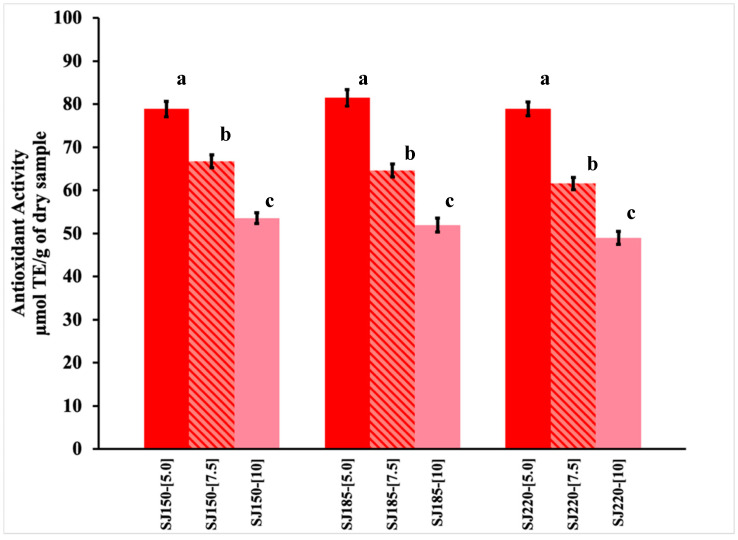
Antioxidant activity of spray-dried SJ-MX powders under different experimental conditions. Inlet Temperatures (150, 185, and 220 °C) and MX concentrations (5%, 7.5% and 10%). a, b and c are parameters of the Tukey’s multiple post hoc tests.

Figure 2 shows the AA of the spray-dried SJ-MX powders obtained at different inlet temperatures and MX concentrations. In general, the AA values varied in the range of 48.95–81.50 μmol of Trolox Equivalents (TE)/g of dry sample for the drying conditions exerted. A similar behavior to TPC was observed for the AA. Significant statistical differences (*p* = 0.05) were observed between SJ-MX powders at 5%, 7.5%, and 10% of MX, where the AA decreased with the increment of the MX concentration. No significant statistical differences (*p* = 0.05) were observed as the inlet temperature increased from 150 to 220.

Martinsen et al. 2020 reported the AA of fresh strawberries (*Fragaria x ananassa* cv Senga Sengana) to prepare jams [18]. They determined the scavenging effect of the strawberry extract towards stable free radical DPPH, indicating a value of 12.6 μmol TE/g of berries, and a high content of ascorbic acid, total monomer anthocyanins, pelargonidin-3-glucoside, and cyanidin-3-glucoside. In the same sense, Alvaréz-Fernandez et al. 2014 studied the elaboration process of strawberry puree [19], stating an antioxidant activity for the mashed strawberries of 23.45 ± 4.01 μmol TE/g of fresh weight. The same authors reported a total of 18 non-anthocyanin compounds in the form of free and conjugated molecules; the most abundant non-anthocyanin compounds quantified were (+)-catechin and HHDP-galloylglucoside. In addition, other flavanols, such as (-)-epicatechin, (-)-epicatechin gallate, and the procyanidin B1, were quantified. p-Coumaroyl hexoside and caffeic acid hexoside were the most abundant hydroxycinnamic acids. The predominant flavonols in these samples were kaempferol. Strawberries are abundant in bioactive compounds with antioxidant properties, like ellagic acid, ellagitannins (ET), anthocyanins, and ascorbic acid. In strawberry fruit, the typical ET is agrimoniin. Research reported that ET, including agrimoniin, possesses antioxidant activity (potent scavenging activity DPPH assays) [20]. All the bioactive compounds reported in the literature can account for the AA found in the strawberry juice powders obtained by the spray-drying procedure using maltodextrin as a carrier agent.

There are different studies on the effect of spray drying of fruit juices on the antioxidant properties of the powders generated. Daza et al. 2017 spray-dried ethanolic extract from cagaita (*Eugenia dysenterica* DC.) using inulin and Arabic gum as carrier agents at 10, 20, and 30%, and evaluated different inlet temperatures (120, 140, and 160 °C) [21]. They reported the highest AA value at 10% Arabic gum and 120 °C (36 ± 1 μmol TE/g DW), and the value decreased as the amount of carrier agent increased at 30% (11 ± 0 μmol TE/g DW). Inulin microcapsules showed lower AA values, and no effect was observed when increasing the temperature from 120 °C to 160 °C, which was associated with the low residence time of the powders in the drying chamber. On the other hand, López-Belchí et al. (2021) spray-dried aqueous extracts from Tintorera grapes (*Vitis vinifera* L.) testing with low and high inlet temperatures (90 and 120 °C) and maltodextrin as carrier agent at concentrations of 10–30% [22]. They reported the highest DPPH+ radical scavenging activity for the powders obtained at 10% MX and low temperature 90 °C (39.97 μmol TE/g DW). However, the AA decreased as the MX content increased to 30% (32.90 μmol TE/g DW) at high temperature. At 120 °C, no significant statistical differences were observed at 10% (34.64 μmol TE/g DW) and 30% (31.18 μmol TE/g DW). Overall, the TPC and AA results indicated that the spray drying of SJ with MX was mainly affected by the concentration of the carrying agent, while barely affected by the inlet temperature. In this sense, the lower MX concentration produced the highest TPC and AA values. The low effect of the temperature suggested that the SJ-MX could be dried at a wide range of temperatures (150–220 °C) without damaging the content of phenolic compounds and antioxidants.

### 2.2. Thermal Characterization

**Figure 3:**
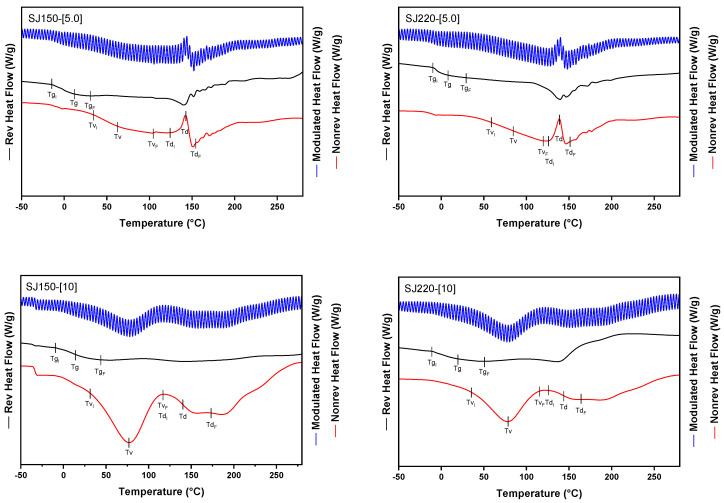
MDSC thermograms of SJ-MX powders prepared at the extreme drying conditions of MX concentration and inlet temperatures.

In complex sugar systems i.e., food products, the identification of different thermal events by differential scanning calorimetry (DSC) is not trivial [23,24,25,26]. Often, the thermal events of the different components overlap, causing an erroneous identification of the event. Therefore, to overcome this problem, the use of modulated differential scanning calorimetry (MDSC) is preferred. The advantage of this technique is the separation of the total heat flow curve into its components, the reversible and non-reversible heat flow curves [26]. Thermal events involving a change in the specific heat capacity i.e., Tg, are observed in the reversible heat flow curve, while those events involving a change in the enthalpy i.e., melting (T_m_) and degradation (T_d_), are observed in the non-reversible heat flow curve. Figure 3 shows the MDSC thermograms of the SJ-MX powders obtained at extreme drying conditions. On each thermogram, three curves are plotted: on top is presented the total heat flow curve (blue color), the reversible heat flow curve is presented in the middle (black color), while the non-reversible heat flow curve is located at the bottom (red color). Additionally, on each curve were identified the beginning, peak, and end of each of the different thermal events. In general, Tg was observed superimposed with first order transitions, such as the evaporation temperature of water (Tv) around 80 °C, the degradation of low molecular weight sugars (glucose, sucrose, fructose) in a range of 141–153 °C, and the degradation of high molecular weight sugars (maltodextrin) in a range of 175–260 °C. The aforementioned thermal events coincide with those reported by Saavedra Leos et al. [27] and [28]. Table 1 summarizes the results of the thermal events determined from the MDCS thermograms for each of the SJ-MX powders. The Tg values increased with both, the concentration of MX and the inlet temperature in the range of 10–24.4 °C. These results were similar to those reported for strawberry and pineapple powdered fruit juices. Gong et al. 2018 reported an increase in the Tg from 32.6 to 38.4 °C when decreasing the concentration of MX in strawberry puree [13]. Nemzer et al. 2018 dried strawberries without using a carrier agent and reported marked differences in the Tg for the strawberry powders dried by the different methods as 23.5 °C for refractance window drying (RWD), 7.2 °C for hot-air drying (AD), and 15.6 °C for freeze-drying (FD) [29]. Hashib et al. 2015 investigated the effect of MX concentration and inlet temperature on the Tg of spray-dried pineapple juice powders. According to them, the increase in the concentration of MX increased the Tg [30]. The rapid formation of a dry surface during the spray drying, inhibits the interactions between the surface of adjacent particles, reducing the plasticizing effect of water and increases the Tg of the system [31,32,33].

**Figure 4:**
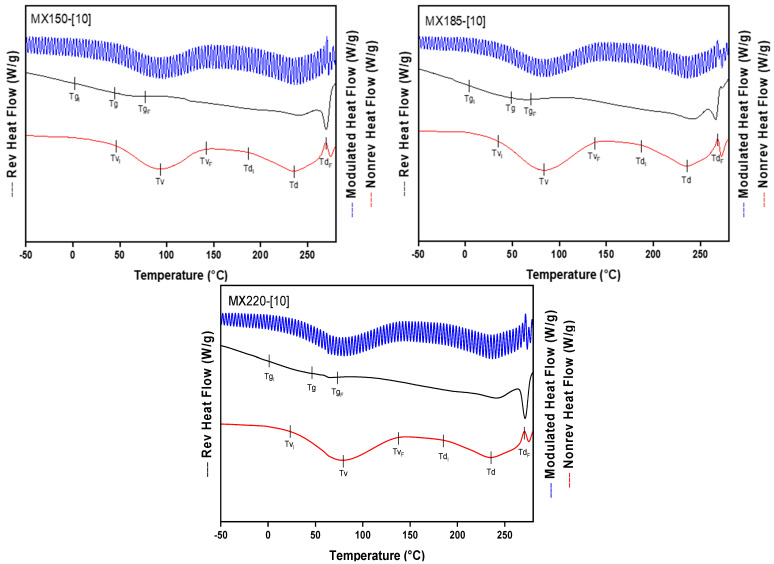
Thermograms of pure MX spray-dried at a concentration of 10%, and different inlet temperatures (150,185 y 220 °C).

In order to properly identifying the different thermal events of SJ-MX, a control sample was also tested. In this sense, pure MX was spray-dried at a concentration of 10% and processed at inlet temperatures of 150, 185, and 220 °C. Figure 4 shows the thermograms corresponding to the dried MXs. The Tg of the control MX is in a range of 55–60 °C, while the degradation temperature (Td) is at 236–240 °C. This suggested that the process temperature did not affect the thermal properties of the MX. These results coincide with those reported by Saavedra-Leos et al. 2015, who reported a similar Tg value for low molecular weight maltodextrin identified as MX10 and a water activity of 0.3 [27].

**Figure 5:**
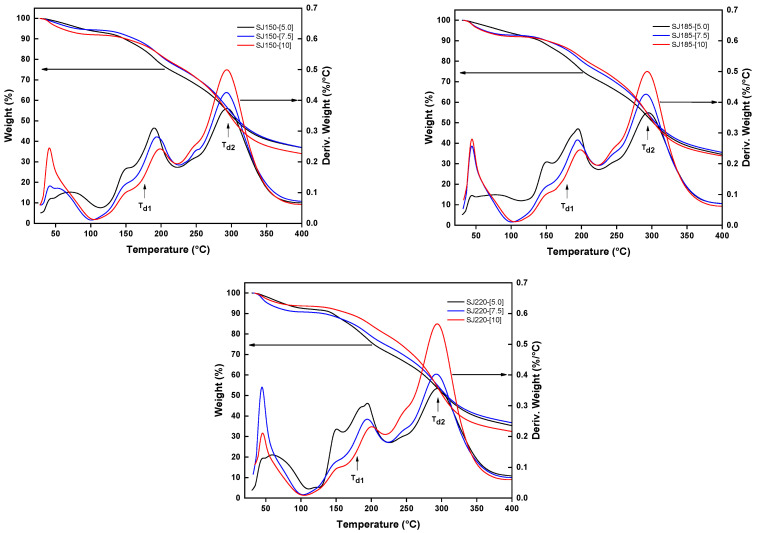
TGA results for all the SJ-MX samples prepared at different spray-drying conditions. Arrows indicate the corresponding axis for TGA (left) and the first derivative of the weight (right).

The thermogravimetric analysis (TGA) quantifies the mass loss as the temperature increases and it is used to determine the thermal stability of food products. Food thermal properties such as water evaporation and thermal decomposition can be established through this analysis [34,35,36]. Figure 5 shows the TGA results for all the SJ-MX samples prepared at the different spray-drying conditions. In general, three thermal events were identified. The first event is observed in a temperature range of 50–150 °C, and it was related to evaporation of water (T_e_) with a mass of 8%. Although the powders were dried, the spray drying process does not remove all of the water, leaving a small amount microencapsulated within the powder particle. Additionally, the powders may have adsorbed a small amount of moisture from the environment. A second thermal event occurred in the range of 150–220 °C with a mass loss about 20%, which may be related to the degradation (T_d1_) of low molecular weight carbohydrates contained in the SJ. The third thermal event was presented in a temperature range of 250–360 °C associated with a mass loss of 25%, which was related to the thermal degradation (T_d2_) of high molecular weight carbohydrates i.e., glucose chains in MX.

### 2.3. Microstructural Analysis

**Figure 6:**
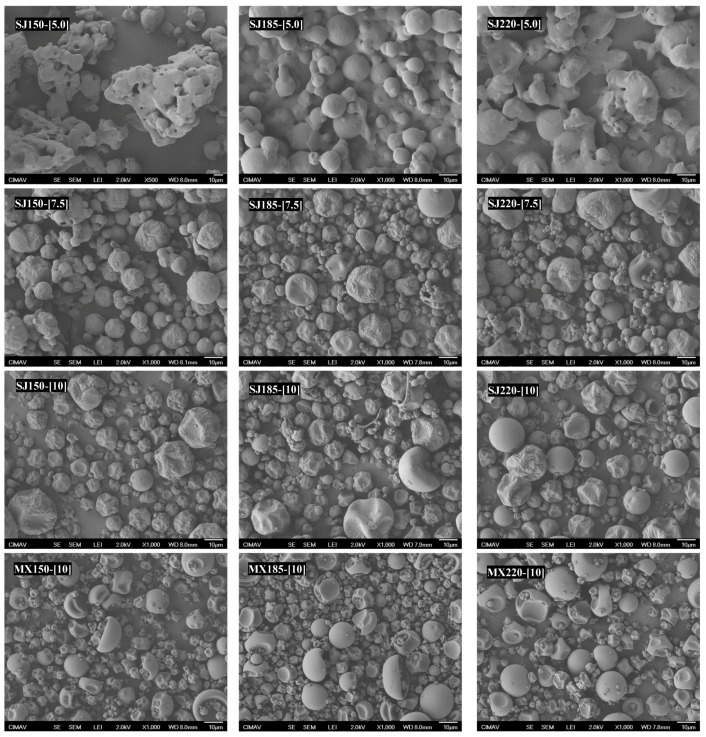
SEM micrograph of SJ-MX powders prepared at the different conditions of MX concentration and inlet temperatures, and of pure MX dried at the different inlet temperatures.

Figure 6 shows the scanning electron microscopy (SEM) micrographs of the SJ-MX powders prepared at the different conditions of MX concentration and inlet temperature. Microscopically, several differences were observed on the morphology of the particles. At the low MX concentration (5%), the particles were observed as aggregated. As the inlet temperature increased, the aggregates did not disappear, but the morphology of the particles was of irregular spherical particle with a smooth surface. With the increment of the MX concentration (7.5 and 10%), the particles were observed well separated and without forming aggregates. However, the increase in the MX concentration affected the surface of the particles, observed as rough in some of the particles, while other were observed as deflated balls. For comparison purposes, the micrographs of the pure MX samples dried at the different temperatures were also included in Figure 6. The morphology, size, and appearance of these particles were similar to those observed for the SJ-MX samples prepared at 7.5 and 10% of MX concentration. These observations suggested that the powder microstructure was better conserved at the intermediate and highest MX concentration. Saavedra-Leos et al. 2021 and Araujo-Díaz et al. 2017 indicated that agglomerated structures are related to a collapse of the microstructure during spray drying [10,15]. Przybył et al. 2018 reported the morphology of strawberry powders—with different quality, the bad batches presented large particle sizes and conglomeration of particles, while the good batches were presented as spherical with deflated shape [37]. Gong et al. 2018 reported the morphology of powdered strawberry juice obtained by spray drying as spherical with smooth surfaces, while increasing the whey protein concentration caused modification of the morphology of the particles and agglomeration [13].

### 2.4. Microestructural Analysis by X-ray Driffraction (XRD)

**Figure 7:**
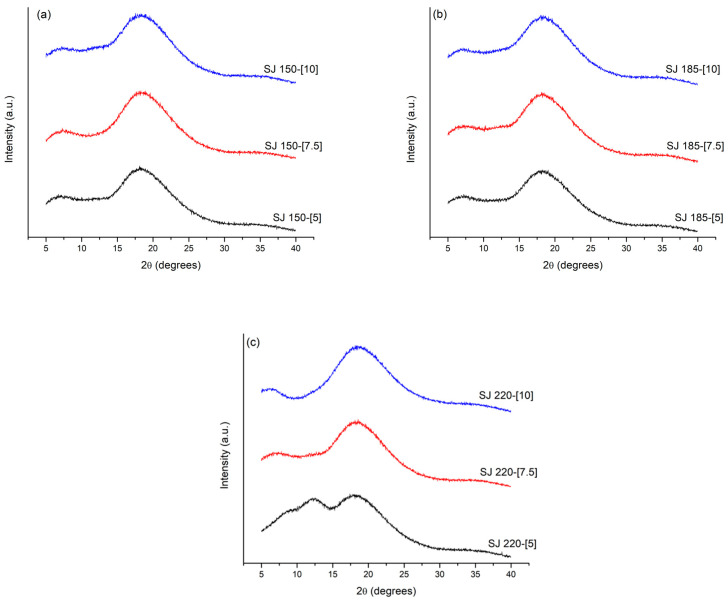
XRD diffractograms of SJ-MX powders prepared at the different conditions of MX concentrations. (**a**) 150 °C, (**b**) 185 °C, and (**c**) 220 °C. The corresponding MX concentration is indicated on each diffractogram.

Figure 7 shows the XRD diffractograms of the SJ-MX powders prepared at the different conditions of MX concentration and inlet temperatures. The diffractograms presented a low intensity broad peak at a diffraction angle about 18°. These observations are characteristics of the amorphous state or low-range order microstructure presented by the MX employed as a carrying agent. As previously reported by Saavedra-Leos et al. 2015, the glucose chains in MX does not present a phase change with the adsorption of water, but a change in the microestructural state from amorphous into rubbery [27]. The advantage of the amorphous state is its high viscosity, which reduces the molecular mobility, limiting the chemical and biochemical reactions. On the other hand, the absence of well-defined diffraction peaks indicated that the microencapsulated components i.e., sugars and bioactive components of the SJ did not form other phases such as individual crystalized particles, but remained microencapsulated within the MX particles [15].

## 3. Materials and Methods

### 3.1. Materials

Strawberries were purchased in a local market in San Luis Potosi, Mexico. Commercial maltodextrin (MX) extracted from cornstarch was acquired from INGREDION Mexico (Guadalajara, Mexico). The dextrose equivalent (DE) of MX was 10, corresponding to a molecular weight of 1625 g/mole and a degree of polymerization (DP) of 2–16 units of glucose [31]. Analytical grade 2,2-diphenyl-1-picrylhydrazyl (DPPH), (±)-6-hydroxy-2,5,7,8-tetramethylchromane-2-carboxylic acid (Trolox), gallic acid, sodium carbonate (Na_2_CO_3_), and Folin–Ciocalteu reagent were purchased from Sigma–Aldrich Chemical Co.

### 3.2. Extraction of Strawberry Juice

For the preparation of the aqueous extract, strawberries were washed with purified water. Next, the strawberry calyxes were removed, and the capped berries were air-dried, then ground in a commercial food processor. The mixture was centrifuged at 10,000 rpm for 10 min at 4 °C to separate the fibers from the liquid. Finally, the supernatant was filtered in a vacuum, and the clarified juice was transferred to a plastic bottle.

### 3.3. Preparation of Spray-Dried Powders

Spray-drying was employed in the preparation of strawberry juice (SJ) powders. The spray-drying conditions were modified from those reported by Vazquez–Maldonado et al. [38]. The preparation of the feeding solutions consisted of mixing the desired amount of MX in 200 mL of SJ. Microencapsulation was carried out in a Mini Spray Dryer B290 (BÜCHI, Labortechnik AG, Flawil, Switzerland) under the following operating conditions: feed temperature of 40 °C, feeding flow of 7 cm^3^/min, hot airflow of 28 m^3^/h, aspiration of 70%, and pressure of 1.5 bar. The inlet temperatures were varied as 150, 185, and 220 °C. This wide range of temperatures was selected to avoid the collapse of the microstructure and other unwanted characteristics such as stickiness and agglomeration of the powders. The obtained powders were weighed and labeled according to Table 2. Powders were individually placed in airtight containers and stored in darkness at 4 °C.

### 3.4. Extraction of Bioactive Compounds from the SJ-MX Powder

The strawberry juice powder extract was obtained using the following procedure: 500 mg of SJ-MX powders were dissolved in 3 mL water. After complete dissolution, 7 mL of pure methanol were added. The extraction was conducted under constant stirring for 1 h. After this period, the extracts were centrifuged for 15 min at 11,000 rpm and 4 °C; the supernatant was filtered and stored at 4 °C in amber bottles to protect them from light damage. Next, the precipitate was re-suspended in a methanol solution (70%) and stirred for 1 h, and the extracts were centrifuged, filtered, and mixed with the previous supernatant [10]. Extracts were prepared in duplicate.

#### 3.4.1. Determination of the Total Phenolic Compounds

The total phenolic content (TPC) of compounds in the extracts was determined via the Folin–Ciocalteu method, using gallic acid as the standard. The reaction was carried out with 0.1 mL of the extract, 0.1 mL of Folin–Ciocalteu reagent diluted (1 N), and 2 mL of a 7.5% solution of Na_2_CO_3_ and 2.8 mL water. The absorbance was measured at 750 nm. The TPC was expressed as mg of gallic acid equivalents (GAE) per mass of dry SJ-MX (mg of gallic acid equivalent (GAE)/g of SJ-MX) [16].

#### 3.4.2. Antioxidant Activity by DPPH

The extract samples were measured in terms of hydrogen-donating or radical-scavenging ability using the stable DPPH radical [39]. Briefly, the reaction mixture contained 100 μL of the extract and 3.9 mL of DPPH solution. The absorbance of the reaction mixture was measured at 515 nm against a blank sample containing only methanol. The results were expressed in terms of the trolox equivalents (TE) per mass of dry powder juice (μmol TE/g SJ-MX), and the total equivalence values were calculated using the standard curve of Trolox.

### 3.5. Thermal Analysis

#### 3.5.1. MDSC

A modulated differential scanning calorimeter (MDSC) Q200 (TA Instruments, New Castle, DE, USA) equipped with an RCS90 cooling system was employed for determining the glass transition (Tg). The instrument was calibrated with indium for melting temperature and enthalpy, while sapphire was used as the standard for heat capacity (Cp). Samples of about 10 mg were encapsulated in Tzero^®^ aluminum pans, (TA Instruments, New Castle, DE, USA). Thermograms were acquired at a temperature range of −50 to 250 °C, with a modulation period of 40 s and amplitude of 1.5 °C. Each experiment was repeated three times.

#### 3.5.2. TGA-DSC-SDT

Thermogravimetric (TGA) and differential scanning calorimetry (DSC) analyses were carried out in a simultaneous TGA-DSC SDT Q600 (TA Instruments, New Castle, DE, USA). For the DSC, the baseline was calibrated with indium (melting temperature of 156.6 °C and melting enthalpy of 28.47 J/g). Samples of 10 mg were encapsulated in standard aluminum pans. Thermograms were recorded at a heating rate of 5 °C/min in a range of 25–400 °C using Universal Analysis 2000© software.

### 3.6. Physicochemical Characterization

#### 3.6.1. Scanning Electron Microscopy

Morphological characterization was conducted using a scanning electron microscope (SEM) (JEOL JSM-7401F) operated at an accelerating voltage of 2 kV. Powder samples were first dispersed on graphite conductive tape, then covered with a thin layer of gold nanoparticles utilizing a sputtering to reduce charging effects (Denton Desk II sputter coater, Denton, TX, USA).

#### 3.6.2. X-ray Diffraction

Microstructural characterization was determined by x-ray diffraction (XRD) analysis in an D8 Advance ECO diffractometer (Bruker, Karlsruhe, Germany) equipped with Cu-K radiation (l = 1.5406 Å) operated at 45 kV, 40 mA and a detector in a Bragg-Brentano geometry. Scans were performed in the 2θ range of 5–50°, with step size of 0.016° and 20 s per step.

### 3.7. Statistical Analysis

The reported TPC and AA values represent the mean and standard error of three replicates. In addition, a one-way analysis of variance (ANOVA) was performed to establish a significance level of 0.05, and Tukey’s multiple post hoc tests were used to determine the difference between the means. The statistical analyses were conducted using the IBM SPSS statistics version 21.0 software (SPSS, Inc., Chicago, IL, USA).

## 4. Conclusions

The spray drying of strawberry juice (SJ) using low concentrations of maltodextrin (MX) and relatively low drying temperatures was successfully achieved. The optimal drying condition for the conservation of the bioactive compounds was found by employing MX concentration of 5% and inlet temperature of 185 °C. At these conditions, bioactive components providing TPC and AA properties were successfully microencapsulated. This was because the low molecular weight sugars of the SJ and the glass transition temperature of the SJ-MX powders decreased, when compared to the pure MX powders. The thermogravimetric analysis showed three thermal events—evaporation of water, thermal decomposition of the sugars in the SJ, and thermal decomposition of the high molecular weight carbohydrates in MX. However, morphological analysis by scanning electron microscopy showed that the microstructure of the powders was better preserved at intermediate and high MX concentrations. Microstructural analysis by X-ray diffraction indicated the presence of amorphous state in all the dried samples. This state is preferred for the microencapsulation of bioactive components because it hinders chemical and biochemical reactions, promoting the conservation of the bioactive components within the powder particles. This research contributes to the knowledge on the spray drying of fruit juices, complex sugar-rich systems, and heat sensitive compounds, which can be applied in the development of food and pharmaceutical products.

## Figures and Tables

**Table 1:** Determined Tg values of SJ-MX powders spray-dry at different conditions.

	SJ150-[5.0]	SJ150-[7.5]	SJ150-[10]	SJ185-[5.0]	SJ185-[7.5]	SJ185-[10]	SJ220-[5.0]	SJ220-[7.5]	SJ220-[10]
**Tgi (°C)**	−13.85	−14.96	−8.03	−7.67	−2.33	−8.25	−9.41	−9.96	−11.35
**Tg (°C)**	10	13.06	16.38	10.33	20.39	21	12.5	24.43	21.93
**Tgf (°C)**	28.87	37.19	43.29	25.98	38.2	38.93	25.26	44.96	44.96

**Table 2:** Experimental design employed for drying the SJ-MX mixtures.

Run	MX Concentration (%)	Inlet Temperature (°C)	Identification
1	5.0	150	SJ150-[5.0]
2	5.0	185	SJ185-[5.0]
3	5.0	220	SJ220-[5.0]
4	7.5	150	SJ150-[7.5]
5	7.5	185	SJ185-[7.5]
6	7.5	220	SJ220-[7.5]
7	10.0	150	SJ150-[10]
8	10.0	185	SJ185-[10]
9	10.0	220	SJ220-[10]

## Data Availability

Data is contained within the article.

## Sample Availability

Samples of the compounds are not available from the authors.
